# Is one item enough? Testing the potential utility of single-item assessments of appearance satisfaction across three studies

**DOI:** 10.1186/s40337-025-01468-8

**Published:** 2025-11-23

**Authors:** Marie-Christine Opitz, Niamh Savage, Katie Talbot, Nora Trompeter, Sarah Moody, Nadia Micali, Ulrike Schmidt, Helen Sharpe

**Affiliations:** 1 School of Health in Social Science, University of Edinburgh, Edinburgh, UK; 2 Great Ormond Street Institute of Child Health, University College London, London, UK; 3 Centre for Clinical Brain Sciences, University of Edinburgh, Edinburgh, UK; 4 Centre for Eating and feeding Disorders Research, Mental Health Center Ballerup, Copenhagen University Hospital – Mental Health Services CPH, Copenhagen, Denmark; 5 Centre for Research in Eating and Weight Disorders, Institute of Psychiatry, Psychology and Neuroscience, King’s College London, London, UK; 6 South London and Maudsley NHS Foundation Trust, London, UK

**Keywords:** Body image, Single-item measures, Emerging adulthood, Psychometrics, ALSPAC

## Abstract

**Background:**

While many multi-item measures are available to evaluate an individual’s body image, study demands occasionally require the use of very brief measures, including single-items. However, little is known about how single-items perform relative to multi-item counterparts.

**Methods:**

The present study aimed to evaluate the utility of two single-item assessments of appearance satisfaction, i.e. an individual’s evaluation of their appearance, using a four-step and seven-step Likert scale and drawing on secondary data from three studies: (1) the Avon Longitudinal Study of Parents and Children (ALSPAC) (*N* = 5,952, aged 14), (2) a cross-sectional study of older adolescents primarily with probable eating disorder diagnoses (the STORY study) (*N* = 242, aged 16–25), (3) and a longitudinal study of emergent adults (the COVID study) (*N* = 201 participants aged 18–30). Thus, the present study aimed to examine the concurrent validity (Body Dissatisfaction Scale, children’s mother-reported body dissatisfaction, Body Esteem Scale for Adolescents and Adults – Appearance Subscale), convergent validity (Eating Disorder Examination Questionnaire, Eating Disorder-15, the Patient Health Questionnaire version 4 and 8), and test-retest reliability of single items measuring appearance satisfaction.

**Results:**

In study 1, the single-item measure showed good concurrent validity with the Body Dissatisfaction Scale for both boys and girls, and moderate concurrent validity with mother-reported body dissatisfaction for girls only. In study 2, acceptable – yet lower – convergent validity was established in a female population with probable eating disorders, and strong convergent and concurrent validity in a small healthy control population. In study 3, strong significant convergent and concurrent validity were established for the implemented single-item assessment in emerging adults with and without a self-reported eating disorder diagnosis, as well as acceptable test-retest reliability.

**Conclusion:**

These results provide insights into the utility of two highly similar single-item assessments of appearance satisfaction and give preliminary support for the use of similar measures in research (specifically for female non-eating disorder populations) when necessary.

## Abstract (plain language summary)

Research questionnaires typically contain multiple questions to assess relevant experiences such as those related to body image. However, long questionnaires can be a burden for participants, especially when a study intends to measure various experiences with multiple questionnaires. To address these concerns, the present study aimed to evaluate the utility of a single question for the assessment of appearance satisfaction. Data from three separate studies was used: a study that included adolescents aged 14 and measured their experiences across time, one study including mostly older adolescents with an eating disorder, and a study assessing experiences of young adults with and without eating disorders multiple times across the COVID-19 pandemic. By comparing findings from the single question with longer, well-established body image and mental health questionnaires, the present study found promising results for the use of the single question, especially for girls and people without an eating disorder. Overall, this study suggests that a single-question measure can be a useful research tool, especially for studies involving female participants without eating disorders and when longer surveys are not practical.

## Introduction

Body image is commonly conceptualised as being multifaceted, consisting of evaluative, perceptual, and behavioural components [5]. The vast majority of body image research, however, focuses on evaluative aspects, i.e., the satisfaction and dissatisfaction with the body or certain aspects of appearance [19]. Body dissatisfaction has been shown to be a key risk factor for mental health difficulties, being associated with higher risk for eating disorders [31], increased psychological distress [14], and poorer mental health-related quality of life [24].

There are a wide range of measures available to researchers to assess evaluative body image (for a review, see [18]. Typically, these will include 8–15 items, although some are much longer (e.g., the Body Shape Questionnaire is 34 items; [8], and may be subscales of larger inventories assessing other aspects of body image or related constructs (e.g., disordered eating). Whilst many multi-item measures of body image are well validated [18], study demands occasionally require the use of very brief measures, including single-items. For example, in large population cohort studies designed to measure a full suite of health and lifestyle factors, each construct may be afforded only one item to limit participant burden. Similarly, clinical studies that aim to capture rich repeated measures data may need to have very brief assessments at each timepoint.

Historically, single-item measures have attracted criticism due to limited evidence of psychometric properties and concerns about measurement error [2]. However, more recently there has been an increased use and evaluation of these measures, with evidence of many performing as well as multi-item counterparts [2]. For example, [26] demonstrated that the item “I have high self-esteem” performs nearly identically to the 10-item Rosenberg Self Esteem Scale. Despite a recent call for action on single-item measures [2], little has been done to explore the reliability and validity of single-item measures of appearance satisfaction. Establishing the psychometric properties of single-item measures of body image would facilitate the use of existing datasets where single-item measures have been included, encourage the inclusion of body image measures in large surveys, and generally provide groundwork for future research where time- and space-efficiency of body image measures is paramount.

The current study aimed to explore the potential utility of two highly similar versions of a single-item assessment of appearance satisfaction across three studies: (1) a large longitudinal study of adolescents (ALSPAC cohort), (2) a cross-sectional study of older adolescents primarily with probable eating disorder diagnoses (STORY study), and (3) a longitudinal study of emerging adults (the COVID study). The present study focused on these developmental stages, as adolescence and young adulthood are associated with higher prevalence of body dissatisfaction [4].

## Overview study 1

Study 1 evaluated the utility of a single-item assessment included in the Avon Longitudinal Study of Parents and Children (ALSPAC) [3, 11] to assess its convergent validity, i.e., similarity with theoretically related constructs [9], in an adolescent population.

### Methods study 1

#### Study design

Participants for study 1 were drawn from the ALSPAC birth cohort, which investigates the environmental and genetic factors affecting cohort members’ health and development. For the current study, information from cohort children at age 14 were included if they provided information on body image variables at this data collection point.

#### Sample selection and participant characteristics

Participants of study 1 were children of mothers residing in Avon, UK, with expected dates of delivery between 1 st April 1991 and 31 st December 1992. The initial number of pregnancies enrolled was 14,541, of which 13,988 children were alive at 1 year of age. The total sample size, including a bolster sample recruited when the oldest children were approximately 7 years of age, was 15,447 pregnancies, of which 14,901 children were alive at 1 year of age. Of the original 14,541 initial pregnancies, 338 were from women who had already enrolled with a previous pregnancy, meaning 14,203 unique mothers were initially enrolled in the study. For the present study, only the first registered twin and cohort children whose sex was reported at birth were included. From the eligible participants, 6,127 completed data at the age 14 timepoint, of which 6,002 (98.0%) completed the single-item assessments of appearance satisfaction. As such, the analytic sample consisted of *N* = 6,002 children (56% female, 44% male; 97% White), of which 0.57% reported having received treatment for an eating disorder either currently or in the past.

#### Measures

##### Single-item assessment of appearance satisfaction

At age 14, young people were asked to provide a response to the question “In the past year how happy have you been with the way your body looks?”, rated on a four-point scale (“very unhappy”, “a little unhappy”, “quite happy”, “very happy”), which was originally included in the McKnight Risk Factor Survey [28].

##### Body dissatisfaction scale

Body Dissatisfaction was assessed using the Body Dissatisfaction Scale [29], which asks young people to rate their satisfaction with individual body parts using a 5-point scale from “extremely satisfied” to “extremely dissatisfied”. Based on gender-specific items (reference to “breasts” for girls and reference to “built” for boys), two separate total scale scores were calculated for boys and girls. The internal consistency of this scale was high for both girls (α = 0.90) and boys (α = 0.93),

##### Mother-reported body dissatisfaction

In addition to self-reported data, mothers also completed measures about their children at age 14, including on body image. A single-item question was asked on body dissatisfaction (“Is s/he upset or distressed about her weight or body shape?”), which was rated on a four-point scale (“No, not at all”, “Yes, a little”, “Yes, quite a lot”, “Yes, a great deal”). Mothers also had the option to select “Don’t know”, which was re-coded as missing.

#### Procedures

The study website contains details of all the data that is available through a fully searchable data dictionary and variable search tool (http://www.bristol.ac.uk/alspac/researchers/our-data/*).* Ethical approval for the study was obtained from the ALSPAC Ethics and Law Committee and the Local Research Ethics Committees. Informed consent for the use of data collected via questionnaires and clinics was obtained from participants following the recommendations of the ALSPAC Ethics and Law Committee at the time.

##### Data Preparation and analyses

All analyses for study 1 were conducted using SPSS version 22. Associations were tested separately for boys and girls, due to gender-specific scale scores. Concurrent validity was investigated using bivariate Pearson correlations between the single-item assessment of appearance satisfaction and the Body Dissatisfaction Scale and mother-reported body dissatisfaction. Effect sizes were interpreted as outlined in [6], where *r* ≥ 0.50 indicates a strong effect (*r* ≥ 0.30 = medium, *r* < 0.10 = poor/small). Missing data for adolescent-reported body dissatisfaction was low (2.6%) whereas mother-reported body-dissatisfaction was higher (16.7%). Little’s MCAR test, χ^2^(2) = 40.15, p = < 0.001, showed that missing data were not missing completely at random; multiple imputation was used to account for missing data to reduce biased estimates. Missing data on both adolescent-reported body dissatisfaction and mother-reported body-dissatisfaction was imputed using multiple imputations with chained equations (MICE) with 10 imputations. Child sex and the single-item appearance satisfaction served as auxiliary variables. Descriptive statistics are reported for raw data, while correlations are reported for imputed data.

### Results

#### Descriptive statistics

Descriptive findings are outlined in Table 1. Boys were significantly more satisfied with their body image compared to girls based on the single-item assessment (boys: M = 2.95, SD = 0.81, 95%CI [2.92; 2.98]; girls: M = 2.64, SD = 0.86, 95%CI [2.61; 2.67], d = 0.38, 95%CI [0.32; 0.43]). Similarly, girls showed higher body dissatisfaction than boys on the Body Dissatisfaction Scale (boys: M = 23.20, SD = 8.51, 95%CI [22.86; 23.53]; girls: M = 27.92, SD = 9.31, 95%CI [27.61; 28.24], d = 0.53, 95%CI [0.47; 0.58]), and mother-reported body dissatisfaction (boys: M = 1.22, SD = 0.51, 95%CI [1.20; 1.24]; girls: M = 1.44, SD = 0.65, 95%CI [1.41; 1.46], d = 0.37, 95%CI [0.31; 0.42]).

**Table 1:** Descriptive statistics by sex (Study 1)

	Boys (*N* = 2641)		Girls (*N* = 3361)		Gender differences
	M	SD	M	SD	Cohen’s d
Single-item body satisfaction	2.95	0.81	2.64	0.86	0.38
Body Dissatisfaction Scale	23.20	8.51	27.92	9.31	0.53
Mother-reported body dissatisfaction	1.22	0.51	1.44	0.65	0.37

Descriptives are based on non-imputed data

All variables showed high levels of kurtosis (i.e., leptokurtic). Additionally, mother-reported body dissatisfaction was positively skewed for both boys and girls.

#### Concurrent validity of the single-item assessment of appearance satisfaction

Concurrent validity was observed for both boys and girls on the Body Dissatisfaction Scale (girls: *r*= − 0.59 [− 0.61; − 0.57]; *p* < 0.001; boys: *r*= − 0.40 [− 0.43; − 0.37], *p* < 0.001), however this was only moderate in boys. Moderate – but still promising - concurrent validity for a parent-reported measure was observed for girls with mother-reported body dissatisfaction (*r*= − 0.33 [− 0.36; − 0.30], *p* < 0.001), but not boys (*r*= − 0.22 [− 0.25; − 0.17], *p* < 0.001).

### Discussion study 1

Findings from study 1 indicate good concurrent validity for ALSPAC’s single-item assessment of appearance satisfaction in girls and moderate concurrent validity in boys. This may be due to gender differences in the conceptualisation of appearance satisfaction, as well as due to further psychometric issues in the single-item measure that need to be explored in the future. A strength of this study was the evaluation of the association between parent-reported body dissatisfaction and self-reported appearance satisfaction, demonstrating a comparatively satisfying link between the two assessments.

#### Limitations study 1

Considering previous findings on the role of ethnicity in the context of body dissatisfaction [4], the present study’s main limitation was the lack of diversity within the study population. Finally, study 1 only included the assessment of the relevant items at a single time point, which did not allow for any re-test evaluations.

## Overview study 2

Study 2 was conducted to evaluate the utility of a single-item, which has previously been used as a potential indicator of evaluative body image in the Millennium Cohort Study (MCS; e.g., [27]. More specifically, study 2 explored the convergent validity of this single-item assessment of appearance satisfaction in a sample of older adolescents with and without putative eating disorders.

### Methods study 2

#### Study design

Participants of study 2 were drawn from the STORY study (Characterising Illness Stages and Recovery Trajectories of Eating Disorders in Young People). STORY is a longitudinal observational cohort study which comprises of online psychometric assessments alongside remote measurement technology including wearable devices and ecological momentary assessments. The full study protocol is illustrated in [22]. The present study used data collected during screening (i.e., assessment of demographics) and initial baseline assessments of relevant constructs. Of note, complete data was available for the STORY dataset, as baseline assessments had to be completed to be eligible for the analytic sample.

#### Sample selection and participant characteristics

At the time of analysis, a total of *N* = 242 UK adolescents (aged 16–25 years, M = 20.3, SD = 2.86) participated in the STORY baseline assessment. Most participants in the sample identified as female (87%) and White (75%). As the study was designed to explore eating disorder experiences, 68% of participants met the criteria of an eating disorder diagnosis based on the Eating Disorder Diagnostic Scale (EDDS) [30]. Given that the sample included both those who were treatment-seeking and those not in contact with health services, we used EDDS scores to identify probable current ED diagnoses for consistency across the sample. Due to the small number of non-female participants (*n* = 31), we subsequently report findings only for female participants (*n* = 211), while also differentiating between a non-eating disorder sample (*n* = 66) and an eating disorder sample with probable eating disorder diagnosis (*n* = 145).

#### Measures

##### Single-item assessment of appearance satisfaction

Participants were asked to provide a response to the question “On a scale of 1 to 7 where ‘1’ means completely happy and ‘7’ means not at all happy, how do you feel about the following parts of your life: The way you look?”. This item was taken from the self-report questionnaire used in the MCS, a large nationally representative survey of young people in the UK (MCS, Sweep 6, Young Person Questionnaire MCS-6, Centre for Longitudinal Studies, [35]).

##### Eating disorder examination questionnaire

The Eating Disorder Examination Questionnaire (EDE-Q) is a 28-item self-report questionnaire which assesses the range, frequency, and severity of eating disorder behaviours during the past 28 days [10]. Of relevance in the current study was the total scale, as well as a composite of the subscales on weight and shape concerns. Cronbach’s alpha in the current study was high for both populations for the total scale (α = 0.93 for the probable eating disorder population, α = 0.97 for the community sample) and the subscale on weight/shape concerns (α = 0.91 for the probable eating disorder sample, α = 0.97 for the community sample).

##### Eating disorder-15

The Eating Disorder-15 (ED-15) is a 10-item eating disorder questionnaire, inquiring about eating disorder behaviours and attitudes throughout the previous week using a 7-point Likert scale ranging from “not at all” to “all the time” [32]. Internal consistency for the ED-15 was high in both populations for the total scale (α = 0.88 in the probable eating disorder population and α = 0.96 in the community sample), as well as the relevant weight/shape concerns subscale (α = 0.88 in the probable eating disorder population and α = 0.94 in the community sample).

##### Patient health questionnaire-8

The 8-item version of the Patient Health Questionnaire (PHQ) was implemented in the current study to measure symptoms of depression in the past two weeks [20, 21]. In the current study, the PHQ-8 showed an acceptable internal reliability in both study populations (α = 0.75 in the probable eating disorder population and α = 0.77 in the community sample).

#### Procedures

The STORY study received ethical approval from the London-Bloomsbury Research Ethics Committee in October 2023 and began data collection in January 2024. Participants were sampled from various sites including NHS Eating Disorder Treatment centres, social media, and various charities (e.g., BEAT). All participants were initially directed to an online screening questionnaire which assessed participants’ demographic information (e.g., age, gender, ethnicity), followed by the EDDS to confirm eating disorder diagnoses. Participants were eligible to take part in the STORY study if they were aged between 16 and 25, residing in the UK, able to independently understand questions and instructions required for study tasks, had no severe disabilities or medical conditions, and were currently not pregnant. Participants were categorised into the probable eating disorder group if they had a current eating disorder diagnosis or met the diagnostic cut-off of the EDDS. Once participants were deemed eligible to participate, they were sent a link to an online consent form which was followed by their first online assessment with a completion time of approximately 1.5 h.

### Results

#### Descriptives

An overview of all descriptive findings is presented in Table 2. On the single-item measure, girls with a probable eating disorder reported significantly poorer body satisfaction (M = 5.64, SD = 1.28, 95%CI [5.43; 5.85]), compared to girls without an eating disorder (M = 4.02, SD = 2.16, 95%CI [3.48; 4.55]). For girls without an eating disorder, the distribution of the single item was only slightly negatively skewed. However, in the probable eating disorder group, the distribution was moderately to strongly negatively skewed. Group means across the rest of the measures reflected a consistent pattern. Girls with a probable eating disorder had higher mean values on the EDE-Q total score (probable ED: M = 4.18, SD = 1.18; non-ED: M = 2.23, SD = 1.74), EDE-Q weight/shape concerns score (probable ED: M = 4.63, SD = 1.18; non-ED: M = 2.68, SD = 1.96), ED-15 total score (probable ED: M = 5.41, SD = 1.08; non-ED: M = 3.31, SD = 1.76), ED-15 weight/shape concerns score (probable ED: M = 5.56, SD = 1.18; non-ED: M = 3.31, SD = 1.86), and PHQ-8 (probable ED: M = 14.88, SD = 5.61; non-ED: M = 8.97, SD = 7.31).

**Table 2:** Descriptive statistics by eating disorder status (Study 2)

	Girls with a probable eating disorder (*N* = 145)		Girls without an eating disorder (*N* = 66)		Group differences
	M	SD	M	SD	Cohen’s d
Single-item body satisfaction	5.64	1.28	4.02	2.16	0.91
EDE-Q total	4.18	1.18	2.23	1.74	1.31
EDE-Q weight/shape concerns	4.63	1.18	2.68	1.96	1.21
ED-15 total	5.41	1.08	3.31	1.76	1.44
ED-15 weight/shape concerns	5.56	1.18	3.31	1.86	1.45
PHQ-8	14.88	5.61	8.97	7.31	0.91

EDE-Q: Eating Disorder Examination Questionnaire, ED-15: Eating Disorder 15, PHQ: Patient Health Questionnaire

#### Convergent validity

Within the probable eating disorder sample, the single-item assessment of appearance satisfaction exhibited good convergent validity with the EDE-Q total

(*r* = 0.49, 95%CI [0.36; 0.60]), EDE-Q weight/shape concern subscale (*r* = 0.58, 95%CI [0.46; 0.68]), ED-15 total (*r* = 0.59, 95%CI [0.47; 0.69]), and ED-15 weight/shape concern (*r* = 0.67, 95%CI [0.57; 0.75]). Moderate convergent validity was observed with the PHQ-8 (*r* = 0.50, 95%CI [0.37; 61]).

Within the non-eating disorder sample, the single-item assessment of appearance satisfaction exhibited very good convergent validity with the EDE-Q total (*r* = 0.76, 95%CI [0.64; 0.95]), EDE-Q weight/shape concern subscale (*r* = 0.82, 95%CI [0.72; 0.89]), ED-15 total (*r* = 0.79, 95%CI [0.68; 0.87]), ED-15 weight/shape concern (*r* = 0.82, 95%CI [0.72; 0.89]), and PHQ-8 (*r* = 0.67, 95%CI [0.51; 0.79]).

### Discussion

Findings from study 2 showed that the present single-item assessment of appearance satisfaction demonstrated good convergent validity among female adolescents with and without probable eating disorders. In fact, psychometric properties of the item were stronger in the non-eating disorder sample. This may be due to the item capturing appearance satisfaction, which is likely more common and more varied in girls without eating disorders, compared to those with an eating disorder.

#### Limitations study 2

No boys and other gender identities were included in Study 2, while sample sizes of the probable eating disorder and community sample were limited. Further, as outlined in study 1, the sample was mainly White, meaning measurement invariance based on ethnicity could not be calculated. Finally, study 2 did not include any specific body image scales, limiting the comparative analyses to relevant eating disorder subscales around body appearance concerns rather than body satisfaction.

## Overview study 3

Study 3 (the COVID study) allowed for further longitudinal exploration of the single item’s performance in a highly relevant population, due to its longitudinal study design and targeted recruitment of individuals with self-reported eating disorders. Specifically, we aimed to determine the concurrent validity, convergent validity, and test-retest reliability of this single-item, and to compare its performance to relevant psychometrically sound, ‘gold standard’ measures.

### Methods study 3

#### Study design

Participants of the COVID study were drawn from a longitudinal study exploring the impact of COVID-19 on eating, exercise, and body image (for full study information, see [25]. The present study involved an analysis of four time points, collected two months apart (data collected September 2020 - April 2021).

#### Sample selection and participant characteristics

The COVID study included *N* = 201 participants (M = 23.5 years, SD = 3.5) from a longitudinal study conducted to assess UK individuals’ experiences of eating and exercise behaviours during the COVID-19 lockdown and beyond. To be included in the analytic sample, participants had to (1) be aged 18–30 years (i.e., ‘emerging adults’, [1]) and (2) have completed outcome measures at least once over the four selected time points. The majority of the sample were female (91.5%) and of White ethnicity (93.0%). Considering that body image is experienced differently within men and women [4], we report findings solely based on female participants (*N* = 184) as the number of male participants (*N* = 17) was too small to conduct separate analyses. Furthermore, as study 3 specifically targeted the recruitment of participants with an eating disorder diagnosis, we are presenting findings separately for those self-reporting an eating disorder diagnosis at any of the included assessment points (*n* = 78) and all participants self-reporting not experiencing an eating disorder (*n* = 106).

#### Measures

##### Single-item assessment of appearance satisfaction

As was the case in study 2, participants were asked to provide a response to the question “On a scale of 1 to 7 where ‘1’ means completely happy and ‘7’ means not at all happy, how do you feel about the following parts of your life: The way you look?”.

##### Body-esteem scale for adolescents and adults

Participants completed the appearance subscale of the Body-Esteem Scale for Adolescents and Adults [23]. This appearance subscale consisted of 10 questions, ranked on a scale from 0 (never) to 4 (always) [23]. The internal consistency of this scale was high in the current study (α = 0.94–0.96 across all timepoints).

##### Eating disorder examination questionnaire-short

The Eating Disorder Examination Questionnaire - Short (EDE-QS) was used to assess eating disorder symptomatology [12]. Participants were asked to answer 12 questions on a scale from 0 (‘not at all’) to 3 (‘markedly’). The EDE-QS has demonstrated good psychometric properties among young women [12]. The internal consistency of this scale was high in the current study (α = 0.86–0.89 across all timepoints).

##### Patient health questionnaire

Anxiety and depressive symptoms were measured using the PHQ-4, [20, 21]. Participants rate how frequently they experienced four different symptoms over the past two weeks on a scale from 0 (‘not at all’) to 3 (‘nearly every day’). The PHQ-4 has previously shown to have sound psychometric properties in young adults [17, 20, 21]. The internal consistency of this scale was high in the current study (α = 0.90–0.93 across all timepoints).

#### Procedures

The original study received ethical approval from the University of Edinburgh Ethics Committee in June 2020 [25]. Data collection involved an anonymous online survey, which was advertised via social media and a UK-based eating disorder charity website (BEAT). All UK residents aged ≥ 18 years were eligible to participate. Following completion of the original study, anonymised data were securely retained for future research. Ethical approval for the current analysis was received in April 2022.

##### Data Preparation and analyses

All analyses were conducted using the Statistical Package for Social Science (SPSS) Version 27. Descriptive statistics were explored for all scales. Little’s MCAR test, χ^2^(339, *N* = 184) = 355.69, *p* = 0.26, showed that missing data were missing completely at random; Expectation Maximisation (EM) was used to estimate missing values for final analyses.

To establish concurrent validity, bivariate Pearson correlations were computed between the single-item assessment of appearance satisfaction and the BESAA, i.e., an established multi-item counterpart appearance satisfaction measure. Convergent validity of the single-item was evaluated using bivariate correlations with theoretically-related measures (disordered eating via the EDE-QS and psychological distress via the PHQ-4). To investigate the test-retest reliability of the single-item, two-month test-retest bivariate correlations were completed. All correlations were calculated at each time point and then average correlations were computed across time points. Suggested guidelines for interpreting test-retest reliability were used (excellent *r* > 0.90, good *r* > 0.80, acceptable *r* > 0.70, questionable *r* > 0.60, poor *r* > 0.50 and unacceptable *r* < 0.50; [2].

Finally, we compared the psychometric properties (concurrent validity, test-retest reliability) of the single-item assessment and the more established body image measure (BESAA) by generating confidence intervals round the relevant Pearson correlation coefficients using an online calculator (www.statskingdom.com, n.d.; cf. [13]. An overlap of confidence intervals between relevant associations indicated no significant difference between the single-item assessment and BESAA in each psychometric property.

### Results

#### Descriptive statistics

Table 3 illustrates the descriptive statistics for the single-item assessment of appearance satisfaction, BESAA, EDE-QS, and PHQ-4 for those who reported an eating disorder and those who reported not having an eating disorder. As might be expected, within the former group single-item values were positively skewed, while in the latter we observed a slightly negatively skewed distribution that was approximately symmetrical.

**Table 3:** Descriptive statistics by time point (Study 3)

		Self-reported eating disorder population (*N* = 78)		Non-eating disorder population (*N* = 106)	
	Time Point	M	SD	M	SD
Single-item assessment of appearance satisfaction	1	5.39	1.43	5.19	1.47
	2	5.33	1.40	5.17	1.46
	3	5.36	1.50	5.23	1.59
	4	5.35	1.42	5.12	1.55
Body-esteem scale for adolescents and adults (appearance)	1	1.08	0.91	1.23	1.04
	2	1.13	0.95	1.25	0.99
	3	1.20	0.93	1.23	0.99
	4	1.46	0.70	1.09	0.96
Eating disorder examination questionnaire-short	1	1.47	0.72	1.41	0.79
	2	1.45	0.77	1.33	0.78
	3	1.47	0.77	1.33	0.74
	4	1.46	0.70	1.34	0.72
Patient-health questionnaire-4	1	1.80	0.93	1.67	0.95
	2	1.76	0.87	1.67	0.95
	3	1.68	0.92	1.66	1.00
	4	1.85	0.88	1.71	0.98

#### Concurrent validity, convergent validity, and test-retest reliability of the single-item assessment of appearance satisfaction

Tables 4 and 5 show the psychometric properties of the single-item assessment of appearance satisfaction. Within both sub-samples, the single-item showed a strong concurrent validity with the established, multi-item appearance satisfaction measure (BESAA), and convergent validity with the EDE-QS, and the PHQ-4. Significant positive associations were found between adjacent time-points of the single-item (*r* = 0.84, *p* < 0.001, 95%CI [0.76; 0.90], self-reported eating disorder sample; *r* = 0.79, *p* < 0.001, 95%CI [0.71; 0.85] non-eating disorder sample), indicating an acceptable to good level of two-month test-retest reliability for the item.

**Table 4:** Correlations (r) of the Single-item measure with the BESAA, EDE-QS, and PHQ-4 (Self-reported ED)

		By time point					Average across time points		
		Single-item assessment of appearance satisfaction (*r*)					Single-item measure (*r*, 95% CI)	BESAA (*r*, 95% CI)	Difference between single-item measure and BESAA (*p*)
Psychometric property	Measure	Time point	1	2	3	4			
Convergent validity	Body esteem scale for adolescents and adults (appearance)	1	− 0.79**				− 0.81** [−0.87, − 0.72]	-	-
		2		− 0.78**					
		3			− 0.81**				
		4				− 0.86**			
Concurrent validity	Eating disorder examination questionnaire-short	1	0.66**				0.66** [0.51, 0.77]	− 0.70** [−0.80, − 0.57]	> 0.05
		2		0.74**					
		3			0.56**				
		4				0.69**			
	Patient health questionnaire-4	1	0.59**				0.57** [0.40, 0.70]	− 0.64** [−0.76, − 0.49]	> 0.05
		2		0.54**					
		3			0.50**				
		4				0.64**			
Test-retest reliability		2	0.76**				0.84** [0.76, 0.90]	0.87** [0.80, 0.92]	> 0.05
		3		0.86**					
		4			0.90**				

**p <.001

**Table 5:** Correlations (r) of the Single-item measure with the BESAA, EDE-QS, and PHQ-4 (non-ED)

		By time point					Average across time points		
		Single-item assessment of appearance satisfaction (*r*)					Single-item measure (*r*, 95% CI)	BESAA (*r*, 95% CI)	Difference between single-item measure and BESAA (*p*)
Psychometric property	Measure	Time point	1	2	3	4			
Convergent validity	Body esteem scale for adolescents and adults (appearance)	1	− 0.83**				− 0.83** [−0.88, − 0.76]	-	-
		2		− 0.79**					
		3			− 0.82**				
		4				− 0.87**			
Concurrent validity	Eating disorder examination questionnaire-short	1	0.71**				0.69** [0.58, 0.78]	− 0.75** [−0.82, − 0.65]	> 0.05
		2		0.66**					
		3			0.67**				
		4				0.71**			
	Patient health questionnaire-4	1	0.76**				0.67** [0.55, 0.76]	− 0.66** [−0.76, − 0.54]	> 0.05
		2		0.69**					
		3			0.56**				
		4				0.67**			
Test-retest reliability		2	0.72**				0.79** [0.71, 0.85]	0.91** [0.87, 0.94]	< 0.05
		3		0.79**					
		4			0.87**				

**p <.001

#### Comparison of the psychometric properties of the single-item assessment of appearance satisfaction and an equivalent multi-item measure (BESAA)

For comparison, the convergent validity of the BESAA with the EDE-QS and PHQ-4, as well as its test-retest reliability, are shown in Table 5. These findings show that the single-item assessment of appearance satisfaction performs very similarly to the BESAA in terms of convergent validity; correlation coefficients between these measures and the EDE-QS and PHQ-4 are of similar magnitude and there is consistent overlap in the 95% confidence intervals. Similar findings can be reported for the test-retest reliability in the self-reported eating disorder sample (average single item re-test reliability: *r* = 0.84, *p* < 0.001, 95%CI [0.76; 0.90], average BESAA re-test reliability: *r* = 0.87, 95%CI [0.80; 0.92]). However, in the non-eating disorder population, the 95% confidence intervals between average re-test reliabilities did not overlap, showing a significant difference in values whereby the BESAA had better re-rest reliability compared to the single-item measure (average single item re-test reliability: *r* = 0.79, *p* < 0.001, 95%CI [0.71; 0.85], average BESAA re-test reliability: *r* = 0.91, *p* < 0.001, 95%CI [0.87; 0.94]).

### Discussion study 3

Findings from study 3 demonstrated that, within both a self-reported eating disorder sample and a self-reported non-eating disorder sample, the tested single-item assessment of appearance satisfaction had good concurrent validity with a well-validated evaluative body image measure, namely the Appearance subscale of the BESAA. Furthermore, the single-item showed similar correlations with disordered eating and psychological distress as the BESAA and had acceptable to good test-retest reliability over repeated two-month assessments.

#### Limitations of study 3

Several limitations need to be considered when interpreting the present findings. First, participants were predominantly reported being female and White, meaning findings cannot be generalised to the wider population. Body image constructs vary greatly between sexes, genders, ages, races, and ethnicities [34]. This could be seen in study 1, where the single-item measure performed worse in boys. Thus, we cannot assume measurement invariance of the single-item between populations. Moreover, sample sizes within the two sub-samples were small (*n* = 78 with a probable lifetime eating disorder and *n* = 106 without an eating disorder), meaning further research is needed to evaluate the single-item measure within a more diverse, larger sample. Future research including a larger sample size and more assessment points will be needed to investigate trajectories of appearance satisfaction using the single- versus a multiple-item assessment. Findings from such analyses will provide better insights into the item’s measurement precision over time by considering patterns of change. Finally, participants of study 3 were predominantly recruited using convenience and snowball sampling, which is associated with high risk of selection bias.

## General discussion

Overall, current findings regarding the use of single-item assessments of appearance satisfaction are promising, especially in an emerging adult sample where correlation coefficients were slightly higher than correlations identified in previous single-item evaluations [26]. Notably, the two versions of the single-items assessed within the current studies already feature within the ALSPAC and MCS youth test battery. While ALSPAC includes a large sample of young people originally from the Bristol area, the MCS is a nationally-representative study which collects longitudinal data from nearly 20,000 individuals and enables large-scale analysis of various health-related outcomes [7]. Therefore, present findings regarding the utility of these items could lay foundations for further body image research through analysing existing data.

Future research will need to address potential sex differences in how appearance satisfaction is being evaluated in ultra-brief measures. Interestingly, study 1 identified a stronger association between the single-item assessment and the Body Dissatisfaction Scale for girls than for boys. Sex and gender differences in both body dissatisfaction [15] and body appreciation [16] have been found previously, however, the present study suggested differences in how a general evaluation of young people’s appearance satisfaction related to ratings of specific body parts. This finding might indicate that girls’ appearance satisfaction is more strongly tied to the evaluation of their individual body parts. Considering that both study 2 and 3 included an exclusively female sample with small sample sizes for relevant sub-groups (those with eating disorders vs. those without), as well as a different body image assessment, more research in this area is needed.

Finally, future research will need to explore more complex analysis techniques (such as the examination of trajectories) to investigate similarities and differences in single- versus multi-item assessment in more depth. While current sample-size- and study-design-restrictions did not allow for such complex analyses, approaches such as latent growth modelling would provide valuable information on a single item’s validity and sensitivity over time.

### General limitations

Giving the promising provisional findings here, further research is warranted to explore the psychometric properties of single-item appearance satisfaction assessments in a range of samples that reflect the general population (e.g., ethnically diverse, different age groups, different cultural contexts). Moreover, the single-items tested here focus exclusively on *appearance satisfaction*. Whilst this is a commonly researched aspect of body image, it is far from the full construct. Further exploration of the potential for single-items, or very brief assessments, to accurately capture other aspects of body image (e.g., affective body image) are needed. Finally, whilst we assessed a range of psychometric priorities, these were not exhaustive. For example, future assessments of predictive and discriminant validity will provide more insights into how single-item assessments of appearance satisfaction can contribute to identifying early signs of eating disorder symptomatology (cf. [33]. While the current study designs did not allow for the assessment of discriminant validity, further investigations are needed to test the conceptual overlap of the single item assessments with constructs that should be empirically distinguishable from appearance satisfaction (e.g., body functionality).

### Conclusions

The present study provides preliminary evidence that a single-item may accurately assess adolescents’ and young adult females’ appearance satisfaction comparably to longer, established measures. Given the rising interest in single-item measures [2], this is an important finding, opening the potential to utilise datasets that include these measures, and to design efficient studies where longer, more established batteries are infeasible. Future work exploring the psychometric properties of single-item assessments in a range of samples and contexts, and identifying ideal wording and response formats for different body image facets, will be valuable.

## Data Availability

Access to ALSPAC data is through a system of managed open access (http://www.bristol.ac.uk/alspac/researchers/access/). Data access to the STORY and COVID study is available upon reasonable request.

## Abbreviations

ALSPAC: Avon Longitudinal Study of Parents and Children
BESAA: Body-Esteem Scale for Adolescents and Adults
ED-15: Eating Disorder-15
EDDS: Eating Disorder Diagnostic Scale
EDE-Q(S): Eating Disorder Examination Questionnaire (Short)
MCS: Millennium Cohort Study
PHQ: Patient Health Questionnaire
SPSS: Statistical Package for Social Science
